# Origin of Distinct Insulating Domains in the Layered Charge Density Wave Material 1*T*‐TaS_2_

**DOI:** 10.1002/advs.202401348

**Published:** 2024-05-10

**Authors:** Hyungryul Yang, Byeongin Lee, Junho Bang, Sunghun Kim, Dirk Wulferding, Sung‐Hoon Lee, Doohee Cho

**Affiliations:** ^1^ Department of Physics Yonsei University Seoul 03722 Republic of Korea; ^2^ Department of Physics Ajou University Suwon 16499 Republic of Korea; ^3^ Center for Correlated Electron Systems Institute for Basic Science Seoul 08826 Republic of Korea; ^4^ Department of Physics and Astronomy Seoul National University Seoul 08826 Republic of Korea; ^5^ Department of Applied Physics Kyung Hee University Yongin 17104 Republic of Korea

**Keywords:** band insulator, charge density waves, Mott insulator, scanning tunneling microscopy, stacking order

## Abstract

Vertical charge order shapes the electronic properties in layered charge density wave (CDW) materials. Various stacking orders inevitably create nanoscale domains with distinct electronic structures inaccessible to bulk probes. Here, the stacking characteristics of bulk 1*T*‐TaS_2_ are analyzed using scanning tunneling spectroscopy (STS) and density functional theory (DFT) calculations. It is observed that Mott‐insulating domains undergo a transition to band‐insulating domains restoring vertical dimerization of the CDWs. Furthermore, STS measurements covering a wide terrace reveal two distinct band insulating domains differentiated by band edge broadening. These DFT calculations reveal that the Mott insulating layers preferably reside on the subsurface, forming broader band edges in the neighboring band insulating layers. Ultimately, buried Mott insulating layers believed to harbor the quantum spin liquid phase are identified. These results resolve persistent issues regarding vertical charge order in 1*T*‐TaS_2_, providing a new perspective for investigating emergent quantum phenomena in layered CDW materials.

## Graphical abstract

Scanning tunneling microscopy results highlight the role of vertical charge orders in a layered charge density wave material. Spatially resolved spectroscopic measurements, along with density functional theory calculations, not only demonstrate the lateral coexistence of multiple insulating domains but also reveal the correlation between their electronic properties and stacking configurations.

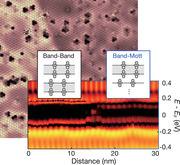

## 1 Introduction

Layered materials exhibit unique electronic properties governed by the atomic and electronic characteristics of their constituent building blocks, as well as their interlayer couplings, including Coulomb interactions, orbital hybridization, charge transfer, and van der Waals forces.^[^ ^1^, ^2^, ^3^ ^]^ Given the susceptibility of these interlayer interactions to various stimuli, such as atomic defects^[^ ^4^ ^]^ and hydrostatic pressure,^[^ ^5^ ^]^ distinct domains with various stacking configurations naturally coexist in layered materials. To understand the electronic properties of these diverse structures and the emergence of exotic quantum phenomena at their interfaces, it is crucial to systematically investigate domain configurations and their electronic structures.

1*T*‐TaS_2_ is an extensively studied 2D correlated system, renowned for its rich phase diagram.^[^ ^5^, ^6^, ^7^, ^8^, ^9^, ^10^, ^11^, ^12^, ^13^ ^]^ The atomic layer is composed of a plane of Ta atoms encapsulated by S atoms with an octahedral coordination. Upon cooling, it undergoes a metal‐insulator transition with a charge density wave (CDW) reconstruction.^[^ ^14^ ^]^ The CDW phase is accompanied by a $\sqrt{13}\times \sqrt{13}$ periodic lattice distortion, comprising clusters that resemble the Star of David (SD). Each SD contains 13 Ta atoms, with 12 forming pairs to create a CDW bandgap, while the unpaired electron of the central Ta atom contributes to a narrow metallic band.^[^ ^6^ ^]^ Due to strong on‐site Coulomb interactions, intralayer electron hopping is strongly suppressed at low temperatures. Since the localized spins of the correlated insulator are frustrated by the triangular lattice, this material is an ideal platform to realize the quantum spin liquid (QSL) phase.^[^ ^15^, ^16^ ^]^

In bulk 1*T*‐TaS_2_, the electronic structure is significantly influenced by the vertical stacking order,^[^ ^17^ ^]^ resulting in the ground state with a dimerized configuration. Interlayer electron hopping promotes a dispersive metallic band along the stacking direction.^[^ ^18^ ^]^ However, at low temperatures, it exhibits insulating electronic properties with commensurate CDWs. Theoretical calculations associate this with the formation of a dimerized insulating layer where the SD is vertically aligned without a lateral shift.^[^ ^19^, ^20^ ^]^ This dimerized configuration is energetically stable because of the energy gain from bonding‐antibonding hybridization of the unpaired electrons; otherwise, it would be prohibited due to Coulomb repulsion. Dimerized band insulating layers are stacked with a lateral shift in the bulk. This is similar to the Peierls' CDW metal‐insulator transition in 1D half‐filled metallic systems.^[^ ^21^ ^]^

Cleaving this layered material produces two distinct insulating surfaces: Type‐I with a dimerized layer termination and Type‐II with an undimerized layer termination. The two distinct surface terminations can be distinguished by their energy bandgaps,^[^ ^20^, ^22^, ^23^ ^]^ with Type‐I exhibiting a larger bandgap compared to Type‐II. They also exhibit distinct spectral responses to electron doping, featuring a rigid band shift and a spectral weight transfer for Type‐I and Type‐II surfaces,^[^ ^24^ ^]^ respectively. In contrast, subsequent scanning tunneling microscopy and spectroscopy (STM/STS) studies have shown a larger gap spectrum on the surface terminated with a single layer rather than a smaller gap.^[^ ^25^ ^]^ Moreover, the larger gap domain has been suggested to host a QSL phase, as evidenced by the band shift in a magnetic field.^[^ ^26^, ^27^, ^28^ ^]^ These results strongly imply that subsurface stacking configurations, which include stacking faults induced by undimerized layers, are crucial for reconciling the discrepancy in the measured tunneling spectra.^[^ ^23^ ^]^ However, experimental verification of subsurface stacking faults, their distribution, and impact on the electronic structure of the surface remains limited.

In this work, we employ STM and STS measurements to visualize distinct insulating domains that form as a result of the change in stacking configurations across the surface of bulk 1*T*‐TaS_2_. Our observations reveal the coexistence of Type‐I and Type‐II domains near a monolayer step on the topmost layer.^[^ ^22^, ^24^ ^]^ Interestingly, in proximity to the monolayer step, both the upper and lower layers are predominantly occupied by the Type‐I termination, suggesting that it is more energetically favorable for the stacking fault to reside beneath the surface. Furthermore, spatially resolved STS measurements over a large area reveal three distinct Type‐I domains separated by both surface and subsurface domain walls. These distinct Type‐I domains show a rigid band shift and band edge broadening, highlighting the interplay between subsurface stacking configurations and the electronic properties of the surface state. Finally, by combining our high‐resolution STS data with density functional theory (DFT) calculations, we demonstrate how different subsurface stacking orders can lead to the observed electronic structures of the surface.

## 2 Results and Discussion

### 2.1 Insulating Commensurate CDW Phase in 1*T*‐TaS_2_

**Figure 1:**
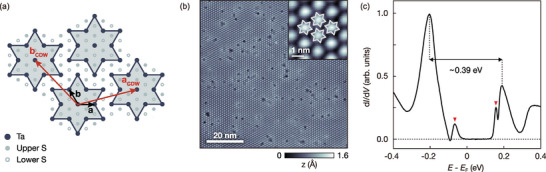
Insulating commensurate CDW phase of 1*T*‐TaS_2_. a) A schematic representation of the atomic structure of 1*T*‐TaS_2_ with the SD superstructure. The black and red arrows correspond to the unit vectors of the 1 × 1 and the $\sqrt{13}\times \sqrt{13}$ lattices, respectively. b) An STM image of the commensurate CDW phase in 1*T*‐TaS_2_ (*V*_set_ = −400 mV, *I*_set_ = 50 pA). Each bright protrusion represents an SD (inset). c) The representative d*I*/d*V* spectrum acquired on the surface of a Type‐I domain (*V*_set_ = −400 mV, *I*_set_ = 50 pA, *V*_mod_ = 5 mV). The bandgap, ≈0.39 eV, is marked by a black arrow. Our spectrum exhibits enhanced band edge features marked by red arrows.

Our data are acquired in the commensurate CDW phase, characterized by the presence of a triangular SD superlattice (**Figure** **1**a). A representative STM image (Figure 1b) shows the SD clusters as bright protrusions (inset). The dark spots in the STM image acquired at −400 mV are intrinsic defects related to sulfur (S) vacancies^[^ ^29^, ^30^ ^]^ whose features are more enhanced at the lower bias due to their in‐gap states (Figure S1, Supporting Information). Figure 1c shows a representative differential conductance (d*I*/d*V*) spectrum acquired at a region away from intrinsic defects. The spectrum displays two prominent peaks located near ±200 meV, which show upward band bending near intrinsic defects or CDW domain walls. These insulating spectral features have been commonly observed in STS measurements.^[^ ^22^, ^24^, ^31^ ^]^ Notably, our high‐resolution STS spectra enable us to finely resolve the band edges as spectral peaks (see red arrows in Figure 1c) with negative differential resistance, which have usually been resolved as shoulders in the tunneling spectra. We attribute the peculiar characteristics to resonant tunneling between localized states of the sample and an atomically sharp STM tip.^[^ ^32^ ^]^ These enhanced spectral features allow us to distinguish domains that have subtle differences in the edges of their electronic bands. The origin of the edges will be discussed in the subsequent section.

### 2.2 Surface Reconstruction in the Layered CDW Material

**Figure 2:**
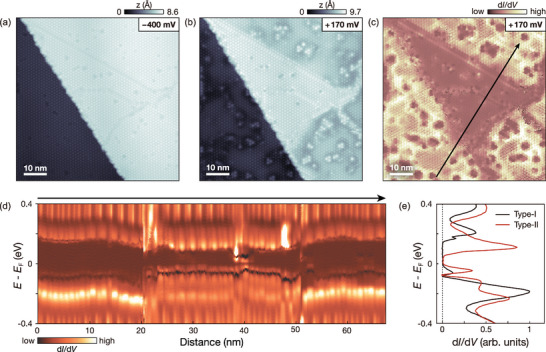
Coexistence of dimerized (Type‐I) and undimerized (Type‐II) layer terminations on the upper terrace of a step edge. a,b) STM images of the surface of bulk 1*T*‐TaS_2_ with the upper and lower terraces separated by a monolayer step obtained at different bias voltages (a *V*_set_ = −400 mV, *I*_set_ = 100 pA and b *V*_set_ = +170 mV, *I*_set_ = 100 pA, *V*_mod_ = 5 mV). c) The d*I*/d*V* map was acquired simultaneously with the STM image shown in b (See Experimental Section). d) Line d*I*/d*V* spectra acquired along the line marked by the arrow shown in (c). e) Averaged d*I*/d*V* spectra of the Type‐I (black) and Type‐II (red) domains acquired from defect‐free areas (*V*_set_ = −400 mV, *I*_set_ = 200 pA, *V*_mod_ = 5 mV).

We first present STM results acquired near a monolayer step to examine the electronic structure on both Type‐I and Type‐II surface terminations. The STM image (**Figure** **2**a) shows bright and dark regions corresponding to the upper and lower terraces, respectively. A subsequent STM image at a reduced bias voltage of +170 mV (Figure 2b) displays a uniform electronic structure across the lower terrace, characterized by a consistent superstructure and absence of domain walls. In contrast, the upper terrace exhibits two types of domains with different electronic structures, separated by surface domain walls. The step height is consistent with the thickness of a single layer 1*T*‐TaS_2_, but is slightly higher at the step edge (Figure S2, Supporting Information). This implies that a higher conductance domain locally forms close to the step edge and rearranges the vertical charge order across the domain wall to maintain a similar electronic structure to the lower terrace.^[^ ^23^ ^]^

The d*I*/d*V* map (Figure 2c), simultaneously acquired with the STM image (Figure 2b), clearly illustrates the presence of two distinct types of domains with varying contrasts on the upper terrace: a dark triangular center domain and bright adjacent domains. Notably, the contrast of the bright domains on the upper terrace matches that of the lower terrace. A series of d*I*/d*V* spectra (Figure 2d), taken along the black arrow in Figure 2c, indicates that the bright domains on both terraces indeed share identical electronic structures. Furthermore, it reveals that while both the bright and dark domains are insulating, their spectra exhibit significant differences in terms of bandgap values and peak intensities (Figure 2e): the peak‐to‐peak bandgaps for the two domain types are ≈0.39 and 0.2 eV, respectively. Since the contrast in the d*I*/d*V* map highlights the relative difference in the electronic structures, the domain walls with in‐gap states in larger gap domains are more prominent than those in smaller gap ones (See Experimental Section). A collection of d*I*/d*V* maps obtained near the step edge (Figure S3, Supporting Information) demonstrates that the larger gap spectrum is dominant across the surface, with the smaller gap spectrum confined to small domains near the step edge.

According to previous DFT calculations^[^ ^23^ ^]^ and many‐body theoretical calculations,^[^ ^20^ ^]^ the larger bandgap of 0.39 eV results from interlayer hybridization in the dimerized layer (Type‐I) and the smaller gap of 0.2 eV arises from the correlated Mott gap in the undimerized surface layer (Type‐II). The latter, a half‐filled layer, exhibits correlated insulating behavior with strong on‐site Coulomb repulsion, otherwise, it shows metallic behavior. This correlated insulating layer is expected to have a smaller gap than the band‐insulating layer with an appropriate Coulomb energy, which stabilizes the dimerized configuration. From the characteristic d*I*/d*V* spectra of the two different domains (Figure 2e), we can identify the areas with higher and lower d*I*/d*V* intensities in Figure 2c as Type‐I and Type‐II domains, respectively (See Experimental Section).

The crossover from Type‐I to Type‐II domains within a terrace is incompatible with the dimerization scenario for the insulating ground state in the layered CDW material. Prior STM studies have demonstrated that the undimerized layer is a Mott insulator with a smaller gap^[^ ^22^ ^]^ and exhibits doping‐induced spectral weight transfer instead of a rigid band shift.^[^ ^24^ ^]^ However, subsequent STM results have shown surfaces with a larger gap spectrum, even when the terraces are separated by a monatomic step.^[^ ^25^, ^33^ ^]^ These observations have attracted renewed attention to the underlying physics of the ground state. Importantly, it was predicted that the undimerized layer (Type‐II) is less stable than the dimerized layer at the surface, leading to the surface reconstruction of the CDWs.^[^ ^23^ ^]^ In systems with undimerized layers at the surface, CDW phase shifts occur via the lateral bond‐relaxation of the SD clusters in the second layers. This reconstruction relocates the undimerized layer from the top to the third layer, leaving a dimerized layer on the surface (Type‐I with a subsurface fault). We can interpret this unique reconstruction as the vertical bond relaxation driven by the lateral bond relaxation. The unstable Type‐II surface can only manifest in small domains near surface steps, where electronic structures at the domain boundaries can overcome the surface energy instability within the domain. Our observations, in which most of the surface is populated by the Type‐I domains with the Type‐II domains existing primarily near the step edge, fully corroborate these theoretical predictions (Figure S3, Supporting Information). It is worth noting that our samples were cleaved at room temperature and subsequently cooled to cryogenic temperatures, ensuring that the vertical configuration of CDW stacking is stabilized during the commensurate CDW formation ≈180 K. This strongly implies the presence of buried undimerized layers, which have been identified, within the otherwise fully dimerized ground state configuration.

### 2.3 Spectroscopic Evidence for Buried Mott Insulating Layers

**Figure 3:**
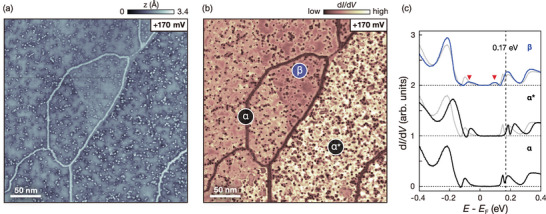
Three different Type‐I insulator domains on a flat surface. a,b) Three different domains are shown by both the a) STM image and b) the d*I*/d*V* map (*V*_set_ = +170 mV, *I*_set_ = 50 pA, *V*_mod_ = 5 mV). c) d*I*/d*V* spectra of the three domains (α, α*, and β) indicated in b averaged over areas not affected by local defects (*V*_set_ = −400 mV, *I*_set_ = 50 pA, *V*_mod_ = 5 mV). Each spectrum is vertically shifted with equal spacing for clarity. The vertical dashed line indicates the energy for the maps shown in (a) and (b). The higher d*I*/d*V* intensity in the tunneling spectra corresponds to the lower contrast in b) the d*I*/d*V* map, which is simultaneously acquired with the topographic image (a) (see Experimental Section) For comparison, the spectrum of domain‐α is superimposed as a faint gray line. There is an upward band shift for domain‐α* and a band edge broadening (marked by red arrows) for domain‐β.

Although the topmost layer is likely to be dimerized, there can be various deviations from the ground state, forming heterogeneous domain configurations.^[^ ^23^, ^34^ ^]^ To demonstrate the presence of distinct stacking orders, we visualize the electronic structure of a large flat terrace without a step edge (**Figure** **3**a). By selecting a bias voltage close to the band edge, we highlight the spatial variation of the band edge that can be influenced by subsurface stacking orders (Figure S4, Supporting Information). Remarkably, the d*I*/d*V* map of the same area (Figure 3b) shows three distinct insulating domains (marked by α, α*, and β) with varying intensities, which up to now have not been thoroughly investigated. The bright (dark) lines in Figure 3a (Figure 3b) represent surface domain walls resulting from CDW phase shifts (Figure S5, Supporting Information). Additionally, the consistency of our tunneling spectra with previous studies,^[^ ^19^, ^22^, ^24^ ^]^ which indicate dimerization of the first and second layers, leads us to attribute the sharp interfaces lacking CDW phase shifts to subsurface domain walls. These domain walls may exist in the third layer or below, giving rise to domains with different stacking orders. Therefore, these domain configurations and their interfaces strongly support the presence of distinct stacking configurations beneath the surface, resulting in a heterogeneous surface electronic structure.

Figure 3c shows the representative d*I*/d*V* spectra of the three distinct insulating domains indicated in Figure 3b. They exhibit the characteristic Type‐I spectrum, implied by the large bandgap of ≈0.39 eV.^[^ ^19^, ^20^, ^22^ ^]^ Despite each having dimerized layers at the surface, there are some clear differences: First, domain‐α* shows a notable rigid band shift of ≈45 meV compared to domain‐α without a significant change in the overall spectral shape. Second, domain‐β exhibits broader band edges (marked by red arrows in Figure 3c) than domain‐α, while the two prominent peaks seem identical. It is worth noting that the variations in the surface electronic structure are not local features caused by defects or domain walls (Figure S6, Supporting Information), as evidenced by the homogeneous background d*I*/d*V* contrast across each domain in Figure 3b.

**Figure 4:**
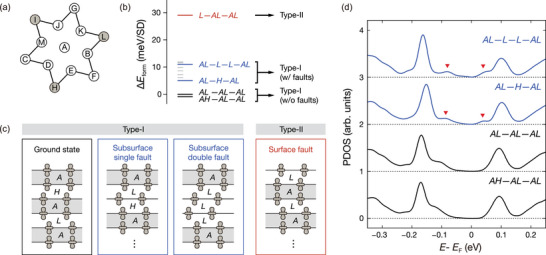
Formation energies and electronic structures of the various stacking configurations. a) A schematic representation of the SD supercell with 13 Ta atoms, each labeled in line with previous studies which have identified *L*, *I*, and *H* (identical by threefold rotational symmetry, shaded in gray) as the most stable sliding configurations among the 12 possibilities (B‐M).^[^ ^19^ ^]^ b) Direct comparison of the formation energies of two different types of stacking configurations and their variations: fully dimerized (black), subsurface undimerized (blue), and surface undimerized configurations (red). Formation energies of other possible stacking configurations, marked by short gray lines, and their corresponding PDOS spectra are presented in Figure S7 (Supporting Information). c) A visual representation of four distinct stacking configurations discussed in (b). Each object denotes the position of the supercell center on individual layers, with the shaded regions (gray) indicating dimerization between adjacent layers. d) The calculated PDOS spectra of the four different Type‐I stacking configurations identified in (b). Each spectrum is vertically shifted with equal spacing, in order of their formation energies, with the lowest at the bottom. The red arrows point to the wider band edges caused by undimerized subsurface layers.

Among the three different Type‐I domains, we observed that domain‐α and domain‐α* appear most frequently on multiple cleaved surfaces. This implies that domain‐α (or domain‐α*) has a lower formation energy compared to domain‐β. To identify the stacking configurations, we adopt the notations from a previous work^[^ ^19^ ^]^ (**Figure** **4**a). Our theoretical calculations clearly show that there are distinct Type‐I configurations with energies lower than the Type‐II (colored red in Figure 4b,c). Note that the layered material likely has all‐dimerized configurations with diverse stacking orders (colored black in Figure 4b,c). In addition, one or more undimerized layers can be found in the bulk,^[^ ^34^ ^]^ leading to a slightly higher formation energy (colored blue in Figure 4b,c). A comprehensive set of calculated formation energies for various stacking configurations is provided in Figure S7 (Supporting Information). Although we cannot precisely determine the number of undimerized layers in the bulk sample, all‐dimerized configurations are likely connected to domain‐α and domain‐α*, while the other Type‐I configurations are associated with domain‐β, with subsurface faults within a few layers close to the surface.

We now focus on the two distinct tunneling spectroscopic features of the surface density of states (DOS) of domain‐α* and domain‐β with respect to domain‐α. The most obvious difference is that domain‐β has broader band edges compared to domain‐α (Figure 3c). Our calculated projected density of states (PDOS) spectra reveal that the spectral features are related to the existence of the undimerized layer underneath the surface (red arrows on blue curves in Figure 4d). The layer‐selective PDOS spectra (See Figure S7, Supporting Information) clearly show that the minor peaks at the band edges in the top‐layer PDOS can be attributed to the coupling with the principal peaks of the third‐layer central Ta atoms. The bandgap for the subsurface dimerized layer is estimated to be 0.18 eV. In contrast, the bandgap of the subsurface undimerized layer is ≈0.11 eV. These results agree with the experimentally observed changes in the gap values of 0.25 and 0.16 eV determined by the minor peaks in domain‐α and domain‐β, respectively.

On the other hand, domain‐α* exhibits spectral characteristics identical to domain‐α with an upward band shift (Figure 3c). These features are well consistent with the calculated spectra for all‐dimerized configurations (black curves in Figure 4c). The band shift is attributed to a higher defect density in domain‐α* compared to domain‐α (Figure S8, Supporting Information). The upward band bending near the intrinsic defects is a typical screening behavior against acceptor‐type dopants in the insulating material.^[^ ^30^ ^]^ Taken together, we conclude that domains with distinct all‐dimerized configurations exist and while their defect densities are not crucial in determining their stacking orders, they do influence their Fermi levels (*E*_F_).

Very recently, magnetic field‐dependent STM experiments^[^ ^26^ ^]^ revealed suggestive fingerprints of the QSL phase predicted to exist in 1*T*‐TaS_2_. Specifically, certain Type‐I spectra exhibited a downward shift in the presence of an external magnetic field, consistent with the theoretical predictions for QSL behavior.^[^ ^27^, ^28^ ^]^ However, two issues must be addressed to establish this phenomenon as direct evidence for the QSL phase. First, it conflicts with the conventional understanding that Type‐I domains do not host localized spins due to interlayer hybridization. Second, some Type‐I spectra do not exhibit the magnetic field response. Our data reconcile these disparate observations by demonstrating the likely presence of Mott insulating layers beneath band insulating surfaces. Thus, the two different behaviors of the Type‐I spectra under a magnetic field can be attributed to subsurface stacking faults, emphasizing that Type‐I domains cannot be characterized solely by a single electronic structure.

## 3 Conclusion

In conclusion, we have identified multiple distinct insulating domains in a wide field of view on the surface of bulk 1*T*‐TaS_2_ with our high‐resolution STS measurements and DFT calculations. We find that the material prefers to maintain a dimerized surface via surface reconstruction and that the subsurface stacking configuration can be altered due to surface reconstruction. The spatial variations in the surface DOS, identified by the reduction of the CDW gap, broadening of the band edges, and a rigid band shift, are revealed to be determined by the differences in stacking configurations and the defect concentration, respectively. These findings provide important insights into reconciling the discrepancies in spectroscopic measurements and identifying this layered CDW material as a platform for a QSL phase. Since our results are related to vertical charge orders, they are envisaged to be highly relevant to many other layered CDW systems, helping to resolve long‐standing open issues about competing phases and ground state formation.

## 4 Experimental Section

### Synthesis and STM Measurements

1*T*‐TaS_2_ crystals (HQ graphene) were synthesized by chemical vapor transport (CVT) with iodine as a transport agent. The 1*T*‐phase was confirmed by low‐temperature micro‐Raman spectroscopy (Figure S9, Supporting Information). Measurements of STM and STS were conducted using a commercial low‐temperature STM (UNISOKU) at a temperature of 4.2 K in an ultra‐high vacuum environment of 1 × 10^−10^ Torr. Mechanically sharpened Pt/Ir (90/10) wires were used for STM tips after characterizing them on a gold (Au) or lead (Pb) (111) surface. Bulk 1*T*‐TaS_2_ samples were cleaved at room temperature and then transferred to the STM head, which had been cooled to 4.2 K. A standard lock‐in technique was used for d*I*/d*V* measurements, with an ac voltage (a voltage modulation amplitude of 5 mV and a frequency of 613 Hz) added to the dc sample bias. Occasionally, d*I*/d*V* maps displayed a reverse contrast to a topographic image, which maps the height of the STM tip with feedback to keep the tunneling current constant. As the STM tip moved away from the surface due to the higher conductance, d*I*/d*V* signal got weaker when it was acquired simultaneously with a topographic image.^[^ ^35^ ^]^

### Density Functional Theory Calculations

DFT investigations were carried out using the Vienna Ab Initio Simulation Package (VASP).^[^ ^36^, ^37^ ^]^ The computational approach involved the projector augmented wave method,^[^ ^38^ ^]^ the generalized gradient approximation for the exchange‐correlation potential,^[^ ^39^ ^]^ and the DFT + *U* formalism as formulated by Dudarev et al.,^[^ ^40^ ^]^ setting the Hubbard *U* parameter to 1.25 eV for Ta 5*d* orbitals. Van der Waals forces were taken into account by applying the Tkatchenko–Scheffler method.^[^ ^41^ ^]^ Electronic wave functions were described with a plane‐wave basis set, with cutoff energies of 323 eV for bulk and 259 eV for surface calculations. Reciprocal space integration was conducted using a 4 × 4 × 8 k‐point mesh within the Brillouin zone of the $\sqrt{13}\times \sqrt{13}\times 1$ supercell. For surface models, a periodic slab geometry with seven or eight layers of TaS_2_ and a vacuum separation of 20 Å was employed. The relaxation of all atomic positions was continued until the forces on each atom were below 0.01 eV Å^–1^.

### Raman Scattering Measurements

Raman spectroscopic experiments were carried out using a λ = 561 nm laser (Oxxius) with the sample mounted onto the cold finger of a He‐flow cryostat (Oxford MicroStat). The laser was focused onto the sample with a spot diameter of 2 µm and a laser power of less than 0.1 mW. Raman‐scattered light was dispersed through a Princeton Instruments Trivista spectrometer onto a charge‐coupled device detector (PyLoN eXcelon).

## Conflict of Interest

The authors declare no conflict of interest.

## Supporting information

Supporting Information

## Data Availability

The data that support the findings of this study are available from the corresponding author upon reasonable request.
