# Supplementary material for: Origin of Distinct Insulating Domains in the Layered Charge Density Wave Material 1T‐TaS2
Source: Adv Sci (Weinh). 2024 May 10;11(28):2401348. doi: 10.1002/advs.202401348 (PMC11267268; doi:10.1002/advs.202401348)
Supplement: Supplementary file 1 — Supporting Information [file ADVS-11-2401348-s001.pdf]

ADVANCED SCIENCE
Open Access

# Supporting Information

for *Adv. Sci.*, DOI 10.1002/advs.202401348

Origin of Distinct Insulating Domains in the Layered Charge Density Wave Material 1*T*-$TaS_2$

*Hyungryul Yang, Byeongin Lee, Junho Bang, Sunghun Kim, Dirk Wulferding, Sung-Hoon Lee* and Doohee Cho**

# Supporting Information for
# Origin of Distinct Insulating Domains in the Layered Charge Density Wave Material $1T$-$TaS_2$

*Hyungryul Yang† Byeongin Lee† Junho Bang Sunghun Kim Dirk Wulferding Sung-Hoon Lee* Doohee Cho**

*†These authors contributed equally to this work.*

Hyungryul Yang, Byeongin Lee, Junho Bang
Department of Physics, Yonsei University, Seoul 03722, Korea

Prof. Sunghun Kim
Department of Physics, Ajou University, Suwon 16499, Korea

Dr. Dirk Wulferding
Center for Correlated Electron Systems, Institute for Basic Science, Seoul 08826, Korea
Department of Physics and Astronomy, Seoul National University, Seoul 08826, Korea

Prof. Sung-Hoon Lee
Department of Applied Physics, Kyung Hee University, Yongin 17104, Korea
Email Address: lsh@khu.ac.kr

Prof. Doohee Cho
Department of Physics, Yonsei University, Seoul 03722, Korea
Email Address: dooheecho@yonsei.ac.kr

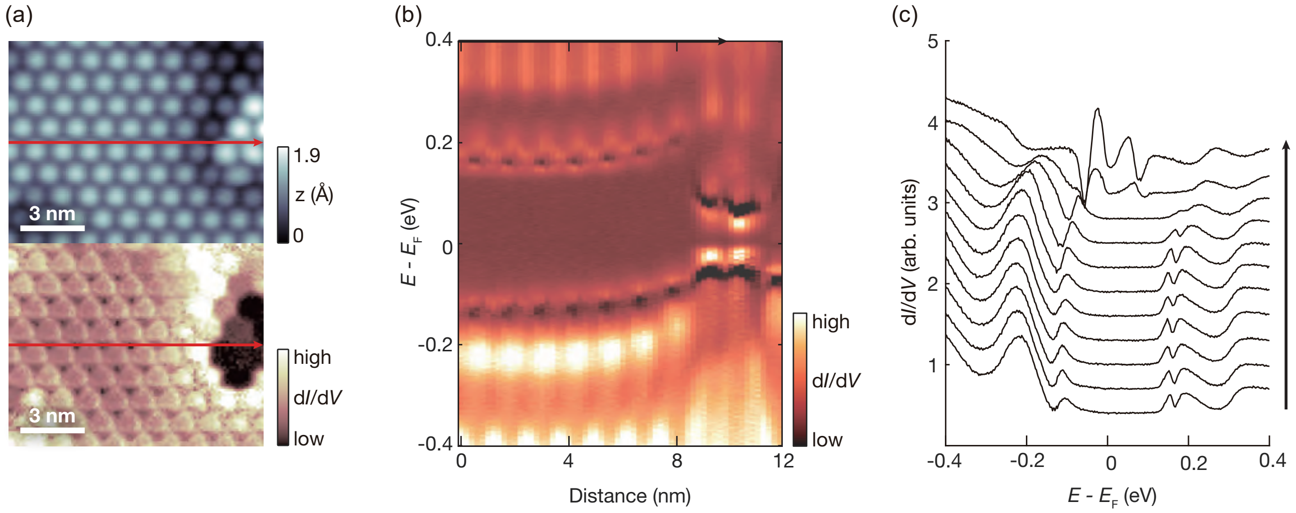


Figure S1. Defect spectra. a) STM image and d$I$/d$V$ map containing defects ($V_{\mathrm{set}}$ = +200 mV, $I_{\mathrm{set}}$ = 50 pA). b) d$I$/d$V$ spectra taken across the red arrows in (a). c) A waterfall plot of the d$I$/d$V$ spectra along the black arrow in (b) with vertical shifts applied for clarity. Near the defects, the spectra show an upward shift in (b-c), which implies that these are acceptor-type defects. On top of the defects, in-gap states appear near the Fermi level ($E_{\mathrm{F}}$).

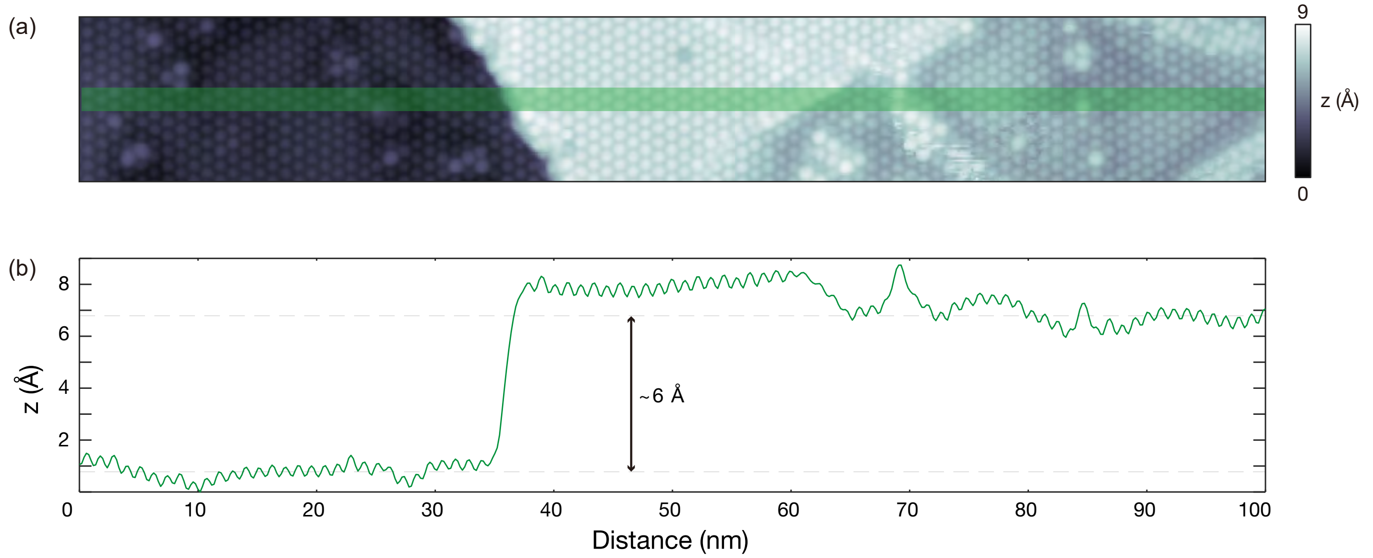


Figure S2. Height of the step edge in Figure 2 of the main text. a) STM image acquired across the step edge shown in Figure 2 of the main manuscript ($V_{\rm set}$ = +170 mV, $I_{\rm set}$ = 100 pA). b) Averaged height profile across the area highlighted in green. A height of around 6 Å is consistent with previous reports of a monolayer step [1, 2]. Type-II surface is slightly higher than the thickness of the monolayer. It undergoes the charge rearrangement accompanied by the surface domain wall (marked by a red arrow), termed surface reconstruction, to become Type-I.

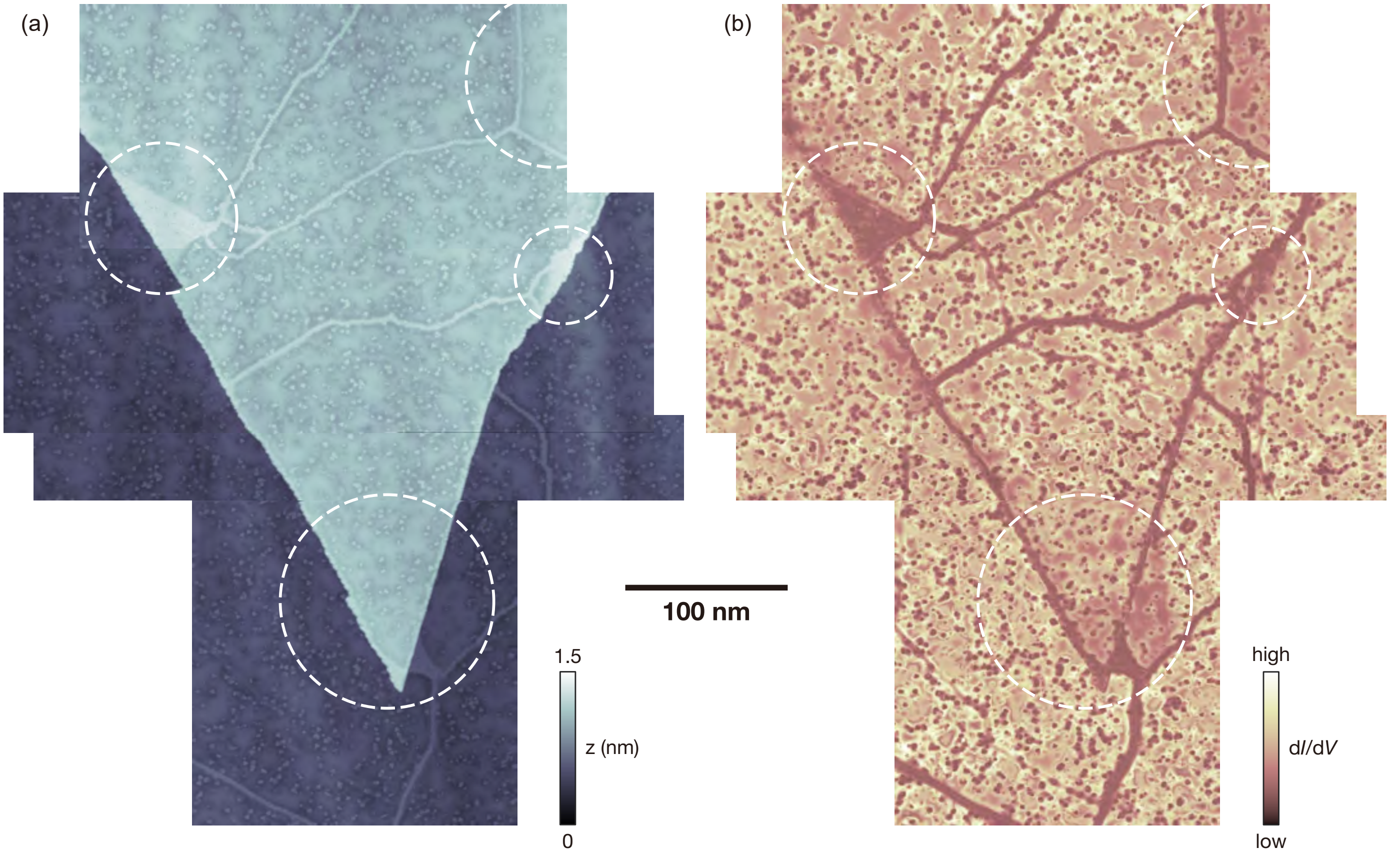


Figure S3. Large field of view of the various insulating states near a monolayer step edge in Figure 2. a-b) Assembled a) STM images and b) d$I$/d$V$ maps show a larger field of view of the area shown in Figure 2 of the main manuscript. We identify various domains, including Type-II and Type-I* ($\beta$), denoted by white dotted circles. However, both the upper and lower terraces are dominated by Type-I domains, which can be seen by the overall homogeneous d$I$/d$V$ intensities ($V_{set}$ = +170 mV, $I_{set}$ = 21 and 30 pA).

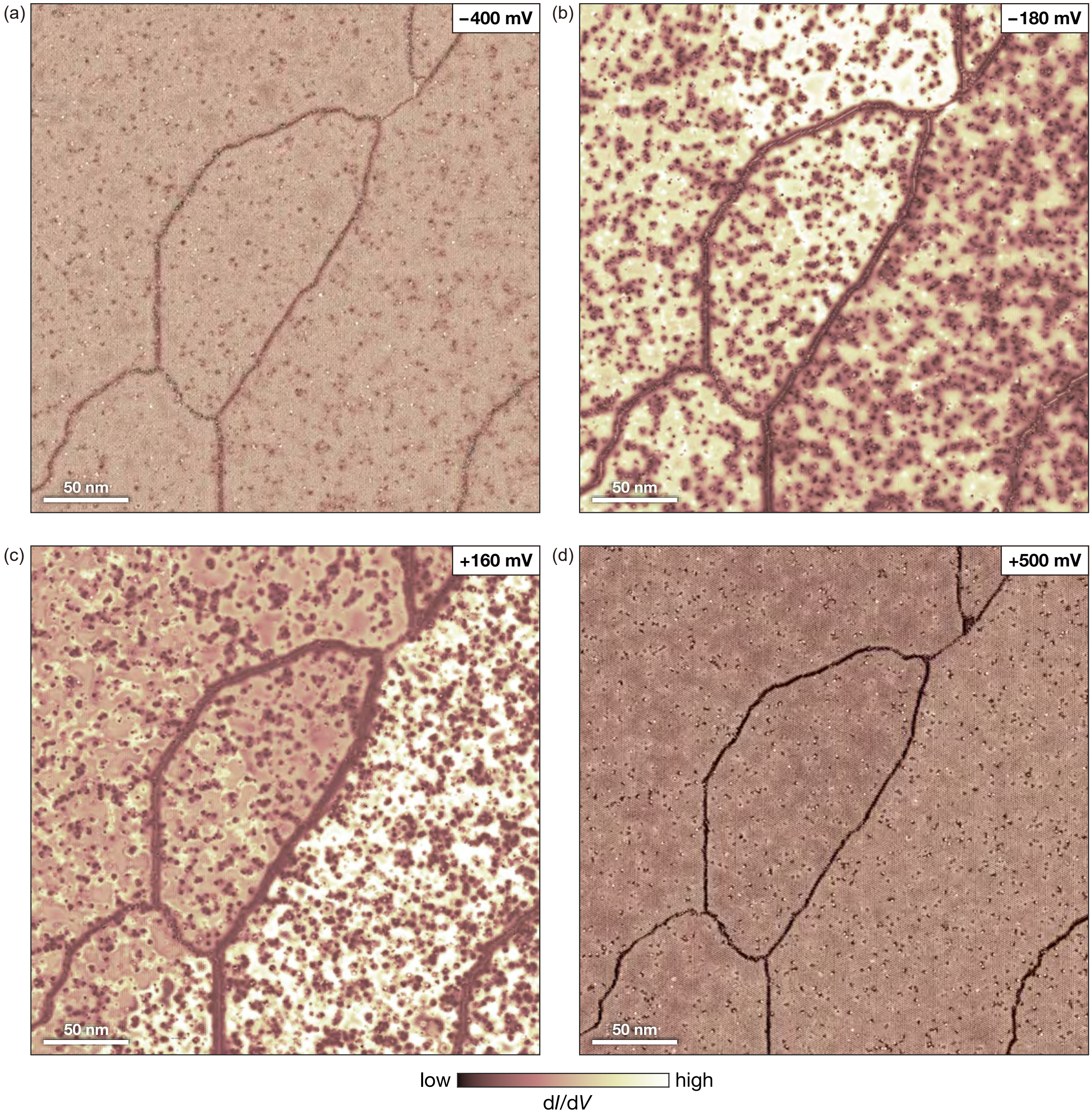


Figure S4. Series of d$I$/d$V$ maps at various bias voltages. a-d) d$I$/d$V$ maps acquired in the same field of view as Figure 3 in the main text at various bias voltages ($V_{set} = -400, -180, +160$ and $+500$ mV, $I_{set} = 50$ pA). The boundaries dividing the domains become more pronounced with lower bias voltages (both positive and negative).

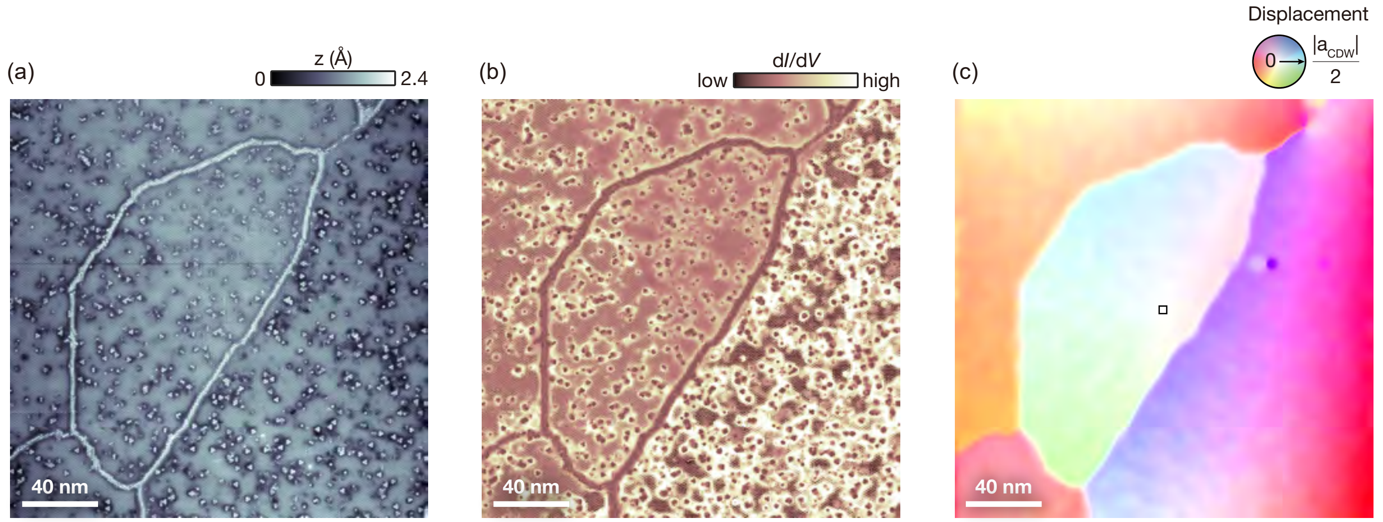


Figure S5. CDW displacement analysis. a-b) Cropped areas of the STM image and d$I$/d$V$ map shown in Figure 3 of the main manuscript ($V_{\mathrm{set}} = +170$ mV, $I_{\mathrm{set}} = 50$ pA). c) CDW displacement map of the same area. We apply the Lawler-Fujita algorithm to analyze the CDW phases across the domain walls, with the black box at the center taken as reference[1, 3]. We identify the presence of abrupt changes in the CDW phases in (c) as surface domain walls, which are seen as bright and dark lines in (a) and (b), respectively.

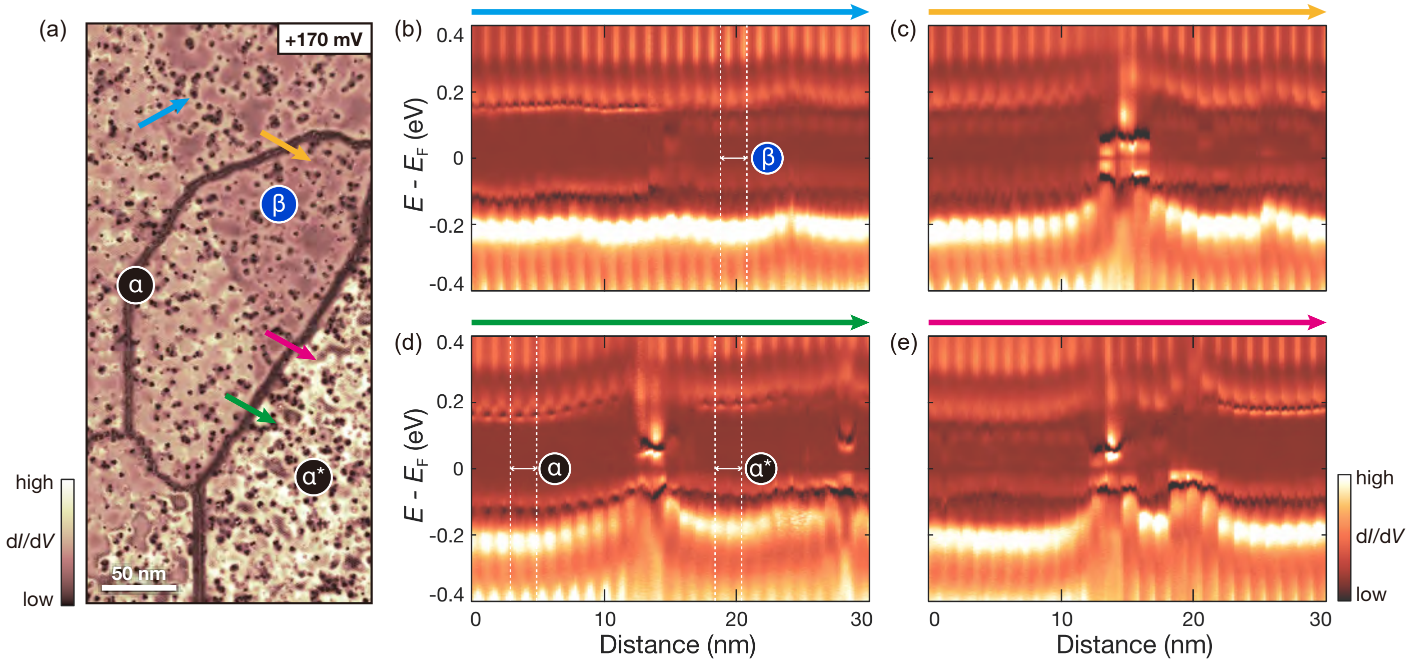


Figure S6. Multiple $\mathrm{d}I/\mathrm{d}V$ spectra taken across the surface and subsurface domain walls shown in Figure 3 in the main text. a) Cropped $\mathrm{d}I/\mathrm{d}V$ map of Figure 3b. b-e) $\mathrm{d}I/\mathrm{d}V$ spectra taken across b) a subsurface domain wall with different electronic structures (blue), c) a surface domain wall with the same electronic structure (yellow), d) a surface domain wall with different electronic structures (green) and e) a surface domain wall with the same electronic structure, continued by a subsurface domain wall with different electronic structures (magenta) ($V_{\mathrm{set}} = -400$ mV, $I_{\mathrm{set}} = 200$ pA). The $\mathrm{d}I/\mathrm{d}V$ spectra for domains-$\alpha$, $\alpha^*$ and $\beta$ in Figure. 3e of the main text are obtained from regions unaffected by local defects and identified in b) and d) as white dotted lines.

(a)

| Stacking | Relative $E_{form}$ | Description |
|---|---|---|
| 7LALALA | 29.1 | Surface fault |
| 8ALHLALA | 11.7 | Subsurface double fault |
| 8AHLHALA | 11.6 | Subsurface double fault |
| 8ALLLALA | 11.0 | Subsurface double fault |
| 7ALLALA | 11.0 | Subsurface single fault |
| 7AIHALA | 9.8 | Subsurface single fault |
| 7AHHALA | 8.6 | Subsurface single fault |
| 7AHLALA | 7.2 | Subsurface single fault |
| 7ALHALA | 5.1 | Subsurface single fault |
| **8ALALALA** | **0.0** | **All-dimerized (reference)** |
| 8AHALALA | -0.8 | All-dimerized |

(b)

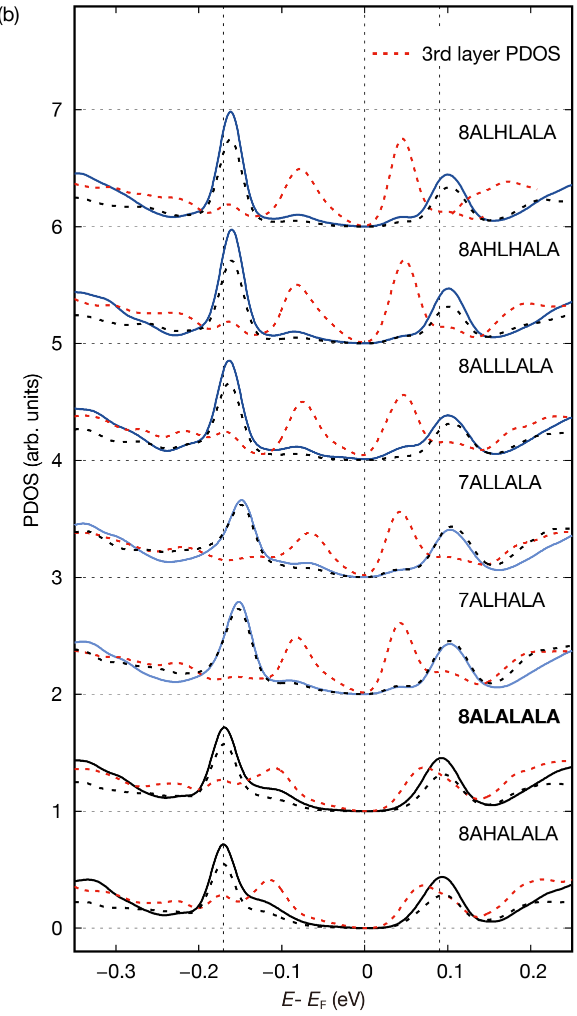


Figure S7. The formation energies of the favorable stacking configurations and their corresponding surface electronic structures. a) Relative formation energies of various stacking configurations, including all-dimerized layers (gray), subsurface single fault (bright blue), subsurface double fault (dark blue) and surface fault (red) with the 8ALALALA stacking configuration taken as the reference point. b) The PDOS spectra of distinct stacking configurations with the layer selective PDOS for the second (black dashed curves) and third layers (red dashed curves). The solid curves correspond to the surface PDOS acquired by integrating $d_{z^2}$ orbital contributions from 13 Ta atoms in the SD cluster.

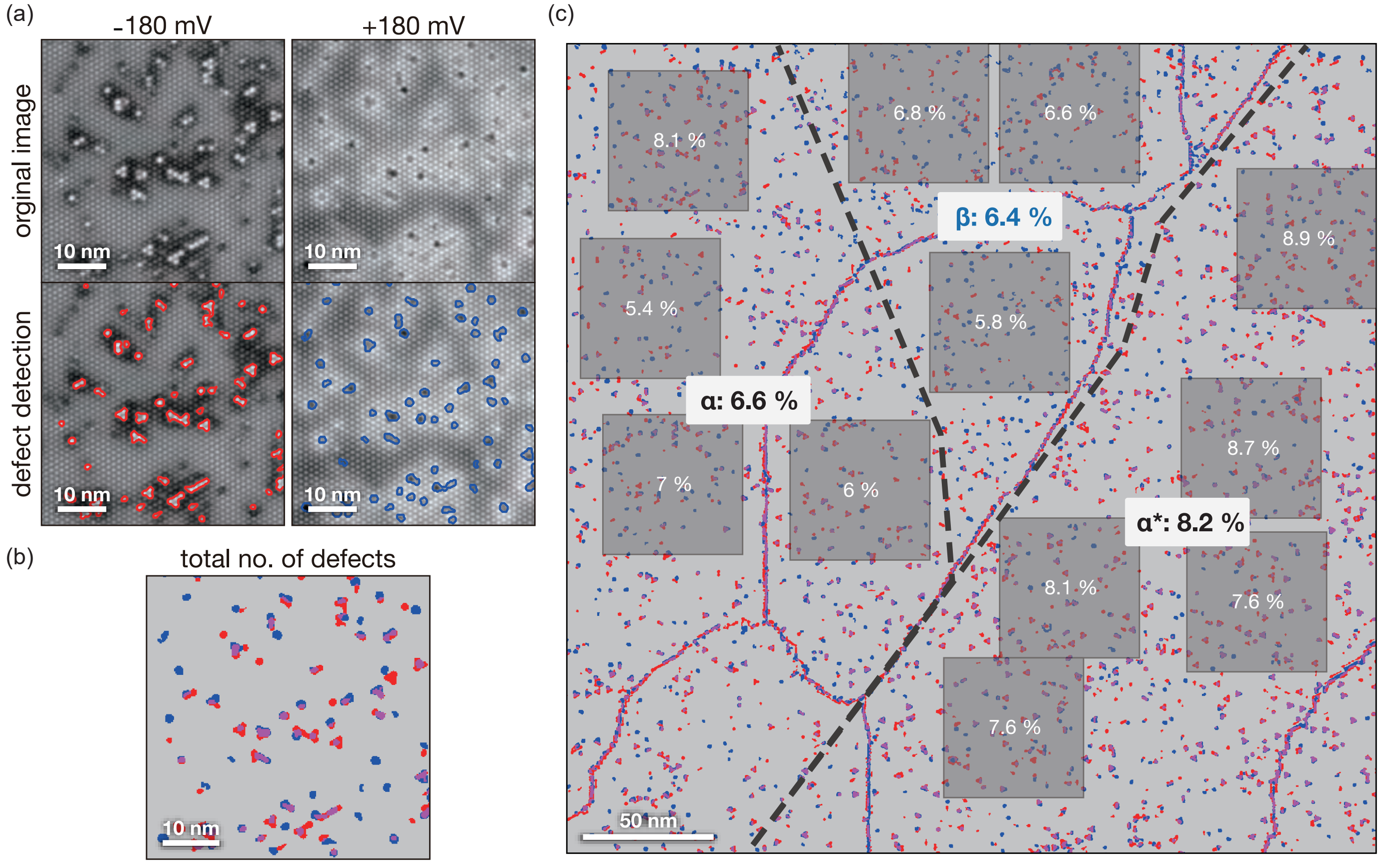


Figure S8. Distribution of defects in each domain. a) STM images extracted from the same location as Figure 3(a-b) at negative and positive bias voltages ($V_{set} = -180$ and $+180$ mV). Defects are highlighted in red and blue for negative and positive bias voltages. At negative bias voltage, defects are usually shown as protrusions in the STM images, whereas at positive bias voltage, they are displayed as depressions. Defect sites in the STM images are identified and marked through image processing techniques. b) Superposition of (red and blue) defects from (a). Some defects are shown only at negative or positive, which are identified as isolated blue or red dots. However, numerous defects are observable at both negative and positive bias voltages, which are shown as purple dots. We quantify the area covered by red, blue and purple to identify defect sites. c) In the individual gray boxes, we note the ratio of defects, while the total averaged values of each domain are shown in the white boxes. Domain-$\alpha^*$ exhibits approximately 1.24 times more defects than the other domains.

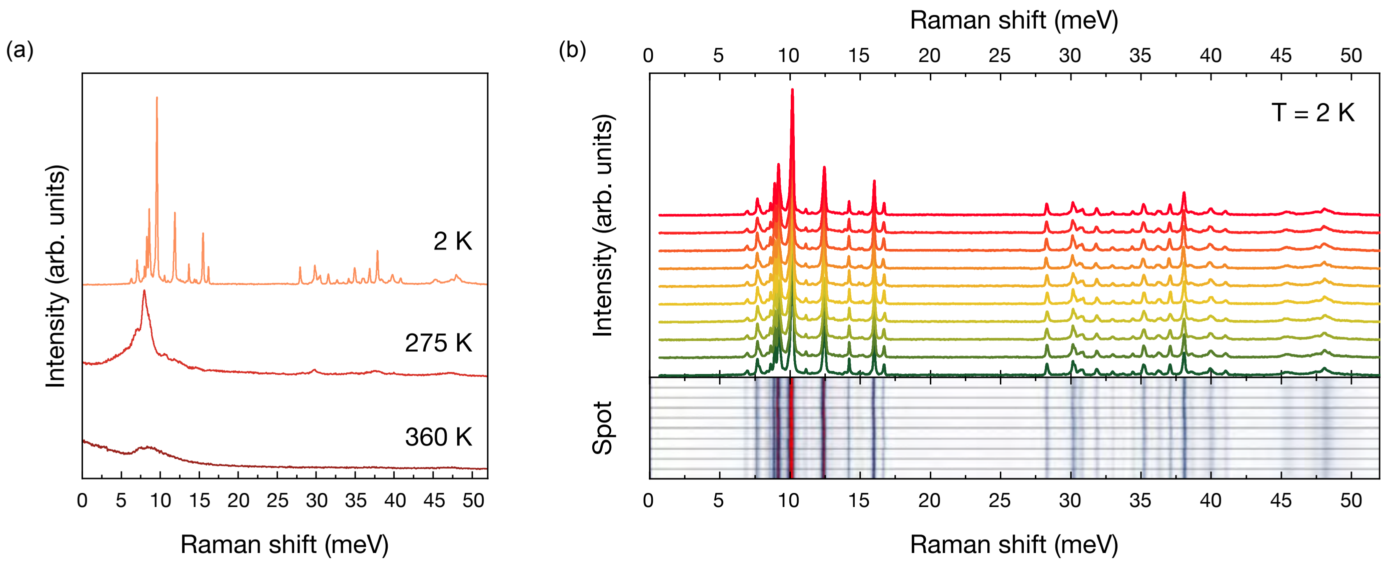


Figure S9. Raman spectroscopy data. a) Raman spectra of $1T$-$TaS_2$, recorded in the ICCDW phase (360 K), the NCCDW phase (275 K) and the CCDW phase (2 K). b) Raman spectra of $1T$-$TaS_2$ in the CCDW phase at $T = 2$ K measured at ten random spots across the mm-sized sample.

Temperature- and position-dependent Raman measurements confirm the global crystalline structure of the sample. Indeed, both the thermal evolution of Raman-active modes through the different CDW phases, as well as Raman spectra taken at ten random spots across the mm-sized sample are in full accordance with the $1T$ phase and clearly distinct from the $4H$ one [4]. We note that with a laser spot diameter of 2 $\mu$m each spectrum contains rather mesoscopic information compared to the truly microscopic nature of the STM study. Interestingly, as can be seen in the color contour plot of panel b), we uncover subtle variations in the form of minor phonon shifts depending on the measurement position, which are greater than the spectral resolution limit of our experiment. While the origin of these variations is currently not fully understood, we interpret them as fingerprints of different Type-I : Type-II ratios, which are expected to slightly modify the energy of CDW-induced zone-folded phonons as well as CDW amplitude modes. We envision that future studies focusing on a detailed Raman mapping through various thermal cycling processes can pinpoint the origin and thereby directly link mesoscopic Raman results to microscopic STM data.

# References

[1] C. Butler, M. Yoshida, T. Hanaguri, Y. Iwasa, *Nature Communications* **2020**, *11*, 1 2477.

[2] J. Lee, K.-H. Jin, H. W. Yeom, *Physical Review Letters* **2021**, *126*, 19 196405.

[3] M. Lawler, K. Fujita, J. Lee, A. Schmidt, Y. Kohsaka, C. K. Kim, H. Eisaki, S. Uchida, J. Davis, J. Sethna, et al., *Nature* **2010**, *466*, 7304 347.

[4] T. Nakashizu, T. Sekine, K. Uchinokura, E. Matsuura, *Physical Review B* **1984**, *29*, 6 3090.
